# Supplementary material for: Strategic attitude expressions as identity performance and identity creation in interaction
Source: Commun Psychol. 2024 Apr 8;2:27. doi: 10.1038/s44271-024-00076-7 (PMC11331991; doi:10.1038/s44271-024-00076-7)
Supplement: Supplementary file 1 — Supplementary Information [file 44271_2024_76_MOESM1_ESM.pdf]

Supplementary Note 1

Ireland, Germany, France, Spain, Albania, Andorra, Austria, Belarus, Belgium, Bosnia and Herzegovina, Bulgaria, Croatia, Czech Republic, Denmark, Estonia, Finland, Greece, Holy See (Vatican City State), Hungary, Iceland, Italy, Latvia, Liechtenstein, Lithuania, Luxembourg, Macedonia, Malta, Moldova, Monaco, Montenegro, Netherlands, Norway, Poland, Portugal, Romania, Russian Federation, San Marino, Serbia, Slovakia, Slovenia, Sweden, Switzerland, Ukraine.

Supplementary Note 2

```
"#boycottRussia lang:en OR #Russiangas lang:en OR #RussianOil lang:en OR \
    #RussianUkrainianWar lang:en OR #StandWithUkraine lang:en OR #StandUpForUkraine
OR \
    #BoycottRussianOil lang:en OR #russianshameday lang:en OR #stoprussia lang:en OR \
    #terrorussia lang:en OR #cancelrussia lang:en OR #SHUTRUSSIADOWN lang:en OR \
    #STOPALLTRADENOW lang:en OR #NoSanctions lang:en OR #dontboycottrussia lang:en
OR \
    #CloseRussianPipelines lang:en OR #UkraineNatoMembershipNOW lang:en OR \
    #CloseUkraineAirSpace lang:en OR #NoFlyZone lang:en OR #NoFlyZoneOverUkraine
lang:en \
    OR #ArmUkraineNow lang:en OR #PlanesForUkraine lang:en OR #NoNuclearWar lang:en
OR \
    #StopSendingWeapons lang:en OR #StopFuelingTheWar lang:en OR \
    #stopfuelingwartwitter lang:en OR #heytwitterstopwarmongering lang:en OR \
    #StopFuelingTheWar lang:en OR #ContainedWarlang:en OR #EndTheWar lang:en OR \
    #NegotiateToDescalate lang:en OR    #StopZelensky lang:en OR #StopTheWar lang:en OR
\
    #PEACE lang:en OR #StopKillingInnocents lang:en OR #VictimsOfViolence lang:en OR \
    #UkraineRussiaWar lang:en OR #NoDoubleStandards lang:en OR #NoSanctions lang:en OR
\
    #NoMilitaryBlocslang:en OR #Peace&SecurityofAllbyAllforAll lang:en OR \
    #SanctionsKILLlang:en OR    #GenocideBySanctions lang:en OR    #Russophobia lang:en
OR    \
    (Support the victims, not perperators) lang:en OR \
    (discrimination against Russians) lang:en OR (ethnic discrimination) lang:en OR\
    (End sanctions against Russia) lang:en OR (blanket rejections to Russian candidates) \
    lang:en OR (Innocent Russian citizens) lang:en OR \
    (Ordinary russians are not responsible for the russian ruling-class) lang:en OR \
    (Discrimination on the grounds of nationality) lang:en OR \
    (Sanctions harm innocent civilians) lang:en"
```

Supplementary Note 3

'#russia', '#ukraine', '#ukraine','#g…', '#ukrainerussiawar', '#ukrainewar', '#russianukrainianwar', '#ukrainian', '#russian', '#ukrainian', '#ukrainerussia', '#g...', '#russiaukrainewar', '#russiaukraine'

Supplementary Note 4

#Genocidebysanction, #sanctionskill, #russiaphobia, #endsanctionsagainstrussia #boycottrussia #sanctionrussia

```
We have  5489 tweets from  3190 unqiue users for the keyword NoSanctions of these, 274 are non-retweets, from 202 unique users
We have  32437 tweets from  13929 unqiue users for the keyword NoFlyZone of these, 20756 are non-retweets, from 6371 unique use
rs
We have  12874 tweets from  5239 unqiue users for the keyword NoFlyZoneOverUkraine of these, 9302 are non-retweets, from 2750 u
nique users
We have  1031 tweets from  616 unqiue users for the keyword NoNuclearWar of these, 360 are non-retweets, from 184 unique users
We have  6396 tweets from  3411 unqiue users for the keyword EndTheWar of these, 3344 are non-retweets, from 1983 unique users
We have  336 tweets from  105 unqiue users for the keyword StopZelensky of these, 275 are non-retweets, from 98 unique users
We have  99700 tweets from  49338 unqiue users for the keyword StopTheWar of these, 38695 are non-retweets, from 17724 unique u
sers
We have  288 tweets from  226 unqiue users for the keyword StopKillingInnocents of these, 49 are non-retweets, from 44 unique u
sers
We have  6336 tweets from  2336 unqiue users for the keyword SanctionsKILL of these, 1283 are non-retweets, from 450 unique use
rs
We have  2898 tweets from  887 unqiue users for the keyword GenocideBySanctions of these, 574 are non-retweets, from 231 unique
users
We have  15604 tweets from  4461 unqiue users for the keyword Russophobia of these, 3456 are non-retweets, from 1958 unique use
rs
We have  148512 tweets from  40588 unqiue users for the keyword ArmUkraineNow of these, 74838 are non-retweets, from 11579 uniq
ue users
We have  1696 tweets from  367 unqiue users for the keyword PlanesForUkraine of these, 1299 are non-retweets, from 249 unique u
sers
We have  109 tweets from  86 unqiue users for the keyword stopsendingweapons of these, 60 are non-retweets, from 55 unique user
s
```

```
We have  12390 tweets from  4042 unqiue users for the keyword  boycottrussia of these, 9696 are non-retweets, from 2743 unique
users
We have  2183 tweets from  1171 unqiue users for the keyword  sanctionrussia of these, 1576 are non-retweets, from 750 unique u
sers
We have  5 tweets from  5 unqiue users for the keyword  nonoflyzone of these, 5 are non-retweets, from 5 unique users
```

Supplementary Note 5

Communities detected in the largest strongly connected component of the directed version of the followership network using the Infomap algorithm as well as how many people shared hashtags from each group.

	Communities						
	0	1	2	3	4	5	6
@#genocidebysanctions	2552	0	2	0	0	0	0

@#sanctionskill	3847	7	490	0	0	0	5
@#cancelhr6600	2815	0	3	0	0	0	0
@#lavrov	12	71	336	12	89	16	68
@#ukraineunderattaÑ□k	1	195	3542	130	309	65	628
@#russophobia	51	161	1458	47	194	171	269
@#boycottrussia	1	466	42	282	930	142	1448
@#stoprussia	5	2030	36	2410	5254	382	5988
@#russiainvadedukraine	0	134	15	278	202	35	628
@#stopputinnow	3	1671	27	499	1234	190	1443
@#nato	21	207	2554	114	331	65	366
#genocideofukranians	0	97	5	920	325	11	375
#peace	753	409	4008	83	227	115	186
@#nebenzia	7	1	165	0	1	4	18
@#stoprussianaggression	0	317	6	653	1135	40	1559
@#ethiopian	813	0	1	0	0	0	1
#russianwarcrimes	2	1610	45	419	788	127	1525
@#kyiv	17	906	2336	654	1060	172	986
#kiev	7	33	2223	7	31	4	45
@#armukrainenow	4	2314	48	2628	6681	202	2490
@#stopwarinukraine	1	77	20	72	292	36	600
@#stopputin	5	541	14	678	1646	277	1737
@#us	462	39	659	48	423	17	115
@#putin	12	886	670	438	1117	260	899

#ukraineunderattack	3	406	99	387	454	104	1302
@#nodoublestandards	235	0	116	0	0	0	2
@#mariupol	14	1158	1780	1183	6688	293	1385
@#russianarmy	1	547	291	476	775	117	1300
@#standupforukraine	0	641	32	683	6411	166	634
@#digitalresistance	0	134	4	9	365	3	1
@#standwithukraine	33	5956	626	5621	11824	1778	7881
@#ethiopia	1198	0	3	0	1	0	0
@#rejects3199	1915	0	0	0	0	0	0

Note. The five most popular hashtags are differentiated by yellow highlight.

Supplementary Note 6

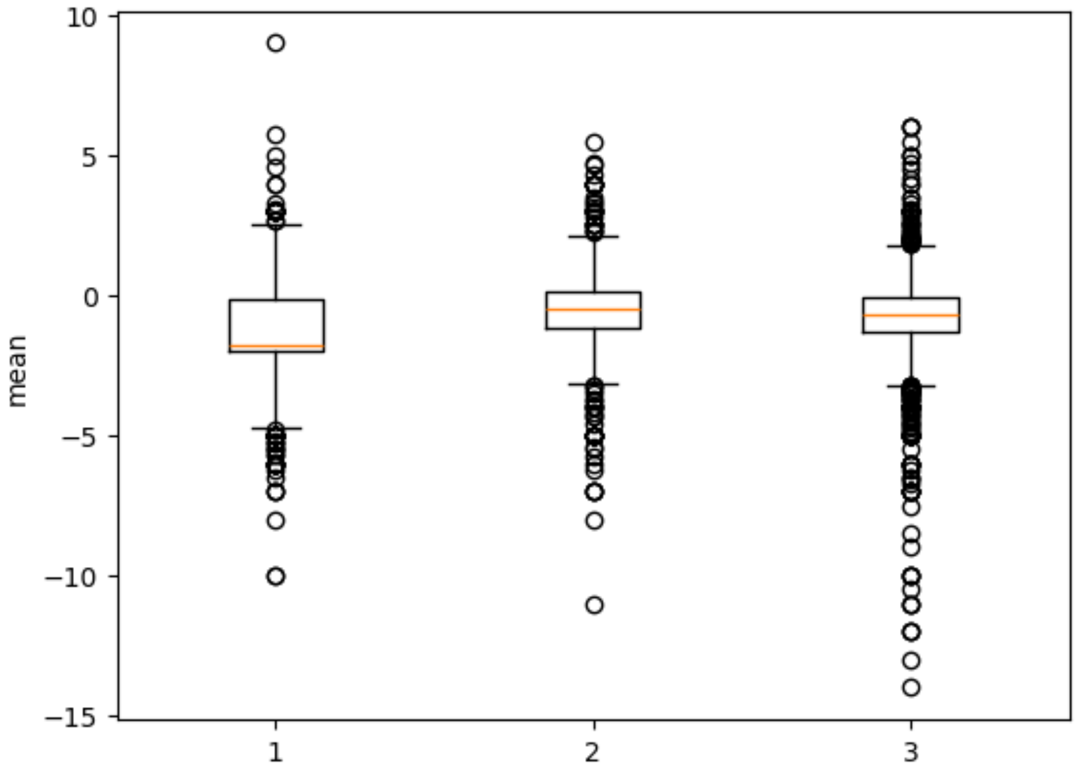

Supplementary Note 7

Tukey pairwise group comparisons between each community

Comparison group	P value	Lower CI	Upper CI
0 v 1	<.001	-1.01	-0.66
0 v 2	<.001	-0.72	-0.42
1 v 2	.001	0.09	0.44
2 v 0	<.001	-0.44	-0.09

Supplementary Note 8

Attitude alignment was highest in the experimental ingroup condition (M = 7.22, SD = 1.73), followed by the control ingroup (M = 6.66, SD = 1.69), the control outgroup (M = 6.18, SD =

1.55) and lowest in the experimental outgroup condition (M = 6.08, SD = 1.68): F (3, 619) = 18.96, $p < .001$ $\eta p2$ = .08. The experimental ingroup condition had significantly higher attitude alignment than all other conditions (the experimental outgroup condition ($p <.001$); the control ingroup condition ($p = .04$); and the control outgroup condition (p <001).

Supplementary Note 9

Time	ANOVA	1 v 2	1 v 3	1 v 4	1 v 5	1 v 6	2 v 3	2 v 4	2 v 5	2 v 6	3 v 4	3 v 5	3 v 6	4 v 5	4 v 6	5 v 6
1	F(3, 1364) = 205.54, p<.001 η_p^2 = .31, 95% CI[.27, .35]	**					**		**	**		**	**			1.00
2	F(5, 1362) = 88.00, p<.001 η_p^2 = .24, 95% CI[.21, .28]	**	**	**	1.00	.07	**	**	**	**	**	**	**	**	**	**
3	F(5, 1362) = 75.87, p<.001 η_p^2 = .22, 95% CI[.18, .25]	**	**	*.31*	*1.00*	*1.00*	**	**	**	**	**	**	**	**	**	**
4	F(5, 1362) = 61.89, p<.001 η_p^2 = .19, 95% CI[.15, .22]	**	**	*	*1.00*	*1.00*	**	**	**	**	**	**	**	**	**	**
5	F(5, 1362) = 85.61, p<.001 η_p^2 = .24, 95%	**	**	**	*1.00*	*1.00*	**	**	**	**	**	**	**	**	**	**

	CI[.20, .27]															
6	*F*(5, 1362) = 71.48, *p*<.001 η_p^2 = .21, 95% CI[.17, .24]	**	**	**	*1.00*	*1.00*	**	**	**	**	**	**	**	**	**	**
7	*F*(5, 1362) = 62.07, *p*<.001 η_p^2 = .19, 95% CI[.15, .22]	**	*.10*	**	*1.00*	*.36*	**	**	**	**	**	*.10*	**	**	*	**
8	*F*(5, 1362) = 41.34, *p*<.001 η_p^2 = .13, 95% CI[.10, .16]	**	**	*1.00*	*.23*	.*	**	**	**	**	**	**	**	**	*	**
9	*F*(5, 1360) = = 56.82, *p*<.001 η_p^2 = .17, 95% CI[.14, .21]	**	**	**	*1.00*	*1.00*	**	**	**	**	**	**	**	**	**	**
10	*F*(5, 1360) = = 38.51, *p*<.001 η_p^2 = .12, 95% CI[.09, .15]	**	**	*1.00*	*.31*	*	**	**	**	**	**	**	**	**	**	*
11	*F*(5, 1360) = = 60.39, *p*<.001 η_p^2 = .18, 95% CI[.14, .22]	**	*	**	*1.00*	*1.00*	**	**	**	**	**	*	**	**	*.10*	**

Note 1. Group 1 = Experimental ingroup incongruence, Group 2 = Experimental ingroup congruence, Group 3 = Experimental outgroup incongruence, Group 4 = Experimental outgroup congruence, Group 5 = Control group incongruence, Group 6 = Control group congruence

*Note 2. ** p* <.001, * *p* <.05

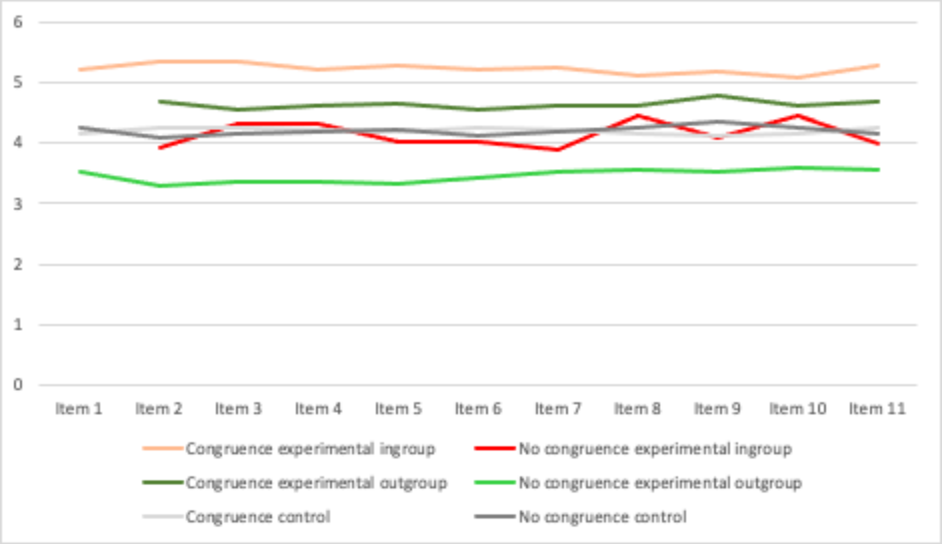

Supplementary Note 10

Materials

Superordinate Opinion-based Social Identification

Participants were instructed to think about other people in society who support Ukraine and asked to rate their sense of identification, solidarity, and strong ties with them. An example is "I identify with other people in society who support Ukraine". All responses ranged from 1 *strongly disagree* to 7 *strongly agree*.

Activism

Activism intentions were measured using the 'conventional activism intentions' questions from the activism orientation scale (Corning & Myers, 2002). An example is "How willing are

you to sign a petition in solidarity with Ukraine?" All responses ranged from 1 *extremely unlikely* to 4 *extremely likely*.

Results

Main Hypothesis: Hypothesis One

We hypothesised that attitude alignment would be greater in the in-group experimental condition compared to the control condition and the out-group condition. We compared how much attitude congruence occurred between dyads in the control group (who did not see each other's attitude answers) to the experimental ingroup (who saw each other's attitudes and began with attitude congruence) and the experimental outgroup (who saw each other's attitudes and began with attitude incongruence). We conducted a one-way ANOVA with Bonferroni post hoc tests, with condition as the independent variable and total attitude congruence as the dependent variable. As hypothesised, attitude alignment was significantly higher in the experimental ingroup condition (M = 7.16, SD = 1.72), compared to the experimental outgroup condition (M = 6.08, SD = 1.70; p < .001, 95% CI [.82, .1.35]) and the control group (M = 6.49, SD = 1.60; p < .001, 95% CI [.41, .94]): F (2, 1363) = 49.86, p < .001, η_p^2 = .07, 95% CI [.4, .09][2]. Thus, hypothesis one was supported. In our preregistration we noted that we suspected outgroup attitude alignment would be lower than the control group. As suspected, the experimental outgroup condition had significantly lower attitude alignment than the control group (p < .001)[3].

Hypothesis Four

We hypothesised that greater congruence would lead to higher activism intentions. We expected congruence to predict activism better in the ingroup condition, versus the outgroup and control condition.

A linear mixed model with the control condition as the comparison group, demonstrated that there was no main effect of total congruence (β = .59, *SE* = .34, *t* = 1.72, *p* = .09, 95% CI [-.08,1.27]), experimental ingroup condition (β = -1.44, *SE* = 2.98, *t* = -.48, *p* =.63, 95% CI [-7.28, 4.41]; *M* = 42.61, *SD* = .59), or experimental outgroup condition (β = -6.12, *SE* = 3.16, *t* = -1.94, *p* = .053, 95% CI [-12.32, .08]; *M* = 43.96, *SD* = .59) compared to the control condition (*M* = 44.05, *SD* = .62). There was no significant experimental ingroup condition by congruence interaction (β = .24, *SE* = .48, *t* = .50, *p* =.62, 95% CI [-.70, 1.19]) or experimental outgroup condition by congruence interaction (β = .75, *SE* = .46, *t* = 1.61, *p* .11, 95% CI -.16, 1.66]). Hypothesis four was not supported.

Hypothesis Five

We hypothesised that greater congruence would lead to greater superordinate attitude based societal identification. We expected congruence to predict superordinate attitude based societal identification better in the ingroup condition, versus the outgroup and control conditions.

A linear mixed model with the control condition as the comparison group, demonstrated that there was a main effect of total congruence (β = .10, *SE* = .03, *t* = 3.03, *p* = .003, 95% CI [.03, .16]). There was no main effect of experimental ingroup condition (β = -.52, *SE* = .29, *t* = -1.79, *p* = .07, 95% CI [-1.08, .05]; *M* = 5.10, *SD* = .05), or control condition (β = .52, *SE* = .27, *t* = 1.93, *p* = .05, 95% CI [-.01, 1.06]; *M* = 5.24, *SD* = .05) compared to the experimental outgroup condition (*M* = 5.24, *SD* = .06). There was no significant experimental ingroup condition by congruence interaction (β = .06, *SE* = .04, *t* = 1.43, *p* <.001, 95% CI [-.02 ,.14]) or control group condition by congruence interaction (β = -.08, *SE* = .04, *t* = -1.87, *p* .06, 95% CI -.17, .01]).

Survey Materials Study Two

Start of Block: Intro

instructions Information Sheet Teamwork & Memory Recall (Ethics Code: 2019_06_19_EHS)

PLEASE READ CAREFULLY

You are invited to take part in a study named ‘Teamwork’. This study is being conducted by X This study is designed to learn more about how encoding information as part of a team can aid memory recall.

You will take part in an online team building exercise with another participant before doing a memory recall task and answering a brief post-survey questionnaire. Full participation will take approximately 10 minutes.

The team building exercise will involve you sharing a brief view on issues with another participant. You will be given a disclosure and more information about the purpose and procedures of the study, once you have completed the study. ·

Identifying information will not be collected from you, but you will be requested to report demographic information such as age and gender.

The data collected may be disseminated but only group-level data will be reported. Confidentiality of your research records will be strictly maintained by X. Each participant's data will remain anonymous.

There are no known risks associated with this study.

Can I change my mind at any stage and withdraw from the study?
Yes. You may decide to withdraw from this research at any time and you will still receive compensation and a full explanation of the study, if you do so.

How will I find out what happens with the project?
You can contact the researcher at X for an update on this project.

Q287 **Before you begin the study please ensure that you understand the following points:**

My participation is voluntary and I may withdraw from the research at any time for any reason.

My participation will involve taking part in a team building exercise and a memory task, before completing a short questionnaire.

I am aware that the team building exercise involves sharing my opinions with another participant in the study

I understand that data will be treated with full confidentiality and, if published, it will not be identified as mine.

I am aware that once data collection is complete I will receive a full explanation of the purpose of the study

Consent I agree to the processing of my data in accordance with the information provided above and I am aware that I may withdraw from the study at any time.

- o Yes (1)
- o No (2)

ProlificID Please enter your prolific ID below

____

Age What is your age?

Gender What is your gender?

- o Male (1)
- o Female (2)
- o Non-Binary (3)

End of Block: Intro

Start of Block: MATCH BLOCK 1

pre-match text

Page Break

match

Waiting for ? participant(s) to join.

match timer Timing

First Click (1)

Last Click (2)

Page Submit (3)

Click Count (4)

Page Break

terminate

End of Block: MATCH BLOCK 1

Start of Block: Block 96

Q292 You have been matched with another participant. This participant has been chosen because they are representative of a particular sample of people.

End of Block: Block 96

Start of Block: Chat block

chat

Remaining time: ${e://Field/remainingTime} seconds Exit chat

Send message

chat end screen Thank you

End of Block: Chat block

Start of Block: letter

Q286 What is your letter?

- o A (1)
- o B (2)

End of Block: letter

Start of Block: Attitude1

Q212 Please note that your answers to all of the questions below will be shared with the other participant

Attitude1 Do you think your country of origin should extend unlimited welcome to as many refugees as need safe refuge?

- o Yes (1)

o No (2)

End of Block: Attitude1

Start of Block: SEND BLOCK 1

send

Page Break

terminate

End of Block: SEND BLOCK 1

Start of Block: GET BLOCK 1

get

Waiting for ? participant(s).

get timer Timing

First Click (1)

Last Click (2)

Page Submit (3)

Click Count (4)

Page Break

terminate

End of Block: GET BLOCK 1

Start of Block: Feedback screen attitude 1

Q116
'Do you think your country of origin should extend unlimited welcome to as many refugees as need safe refuge?'

Participant A has answered ${e://Field/transferA1}

Participant B has answered ${e://Field/transferB1}

End of Block: Feedback screen attitude 1

Start of Block: Identity1

id1a I identify with the sample that my partner is representative of

- Strongly disagree (1)
- Disagree (2)
- Somewhat disagree (3)
- Neither agree nor disagree (4)
- Somewhat agree (5)
- Agree (6)
- Strongly agree (7)

id1b I feel solidarity with the sample

- Strongly disagree (1)
- Disagree (2)
- Somewhat disagree (3)
- Neither agree nor disagree (4)
- Somewhat agree (5)
- Agree (6)
- Strongly agree (7)

id1c I feel strong ties with the sample

- Strongly disagree (1)
- Disagree (2)
- Somewhat disagree (3)
- Neither agree nor disagree (4)
- Somewhat agree (5)
- Agree (6)
- Strongly agree (7)

End of Block: Identity1

Start of Block: Attitude 2

Att2 Many companies have withdrawn from Russia. Some argue that it is necessary to destabilize Russia and undermines Putin's position and control. Others argue that this leaves innocent civilians without employment or access to necessary resources.

Do you think that companies should withdraw from Russia?

- Yes (1)
- No (2)

End of Block: Attitude 2

Start of Block: Send block 2

Q217

Page Break

Q218

End of Block: Send block 2

Start of Block: Get block 2

Q219

Waiting for ? participant(s).

Q220 Timing

First Click (1)

Last Click (2)

Page Submit (3)

Click Count (4)

Page Break

Q221

End of Block: Get block 2

Start of Block: Feedback screen attitude 1, 2

Q140
Do you think that companies should withdraw from Russia?

Participant A has answered ${e://Field/transferA2}

Participant B has answered ${e://Field/transferB2}

Previous attitudes:

'Do you think your country of origin should extend unlimited welcome to as many refugees as need safe refuge?'

Participant A has answered ${e://Field/transferA1}

Participant B has answered ${e://Field/transferB1}

End of Block: Feedback screen attitude 1, 2

Start of Block: Identity2

id2a I identify with the sample that my partner is representative of

- o Strongly disagree (1)
- o Disagree (2)
- o Somewhat disagree (3)
- o Neither agree nor disagree (4)
- o Somewhat agree (5)
- o Agree (6)
- o Strongly agree (7)

id2b I feel solidarity with the sample

- o Strongly disagree (1)
- o Disagree (2)
- o Somewhat disagree (3)
- o Neither agree nor disagree (4)
- o Somewhat agree (5)
- o Agree (6)
- o Strongly agree (7)

id2c I feel strong ties with the sample

- o Strongly disagree (1)
- o Disagree (2)
- o Somewhat disagree (3)
- o Neither agree nor disagree (4)
- o Somewhat agree (5)
- o Agree (6)

- Strongly agree (7)

End of Block: Identity2

Start of Block: Attitude 3

Att3 Do you think NATO’s expansion had a strong influence on Russia’s invasion of Ukraine?

- Yes (1)
- No (2)

End of Block: Attitude 3

Start of Block: Send block 3

Q222

Page Break

Q223

End of Block: Send block 3

Start of Block: Get block 3

Q224

Waiting for ? participant(s).

Q225 Timing

First Click (1)

Last Click (2)

Page Submit (3)

Click Count (4)

Page Break

Q226

End of Block: Get block 3

Start of Block: Feedback screen attitude 1, 2, 3

Q145
Do you think NATO's expansion had a strong influence on Russia's invasion of Ukraine?

Participant A has answered ${e://Field/transferA3}

Participant B has answered ${e://Field/transferB3}

Previous attitudes:

1. 'Do you think your country of origin should extend unlimited welcome to as many refugees as need safe refuge?'

Participant A has answered ${e://Field/transferA1}

Participant B has answered ${e://Field/transferB1}

2. ' Do you think that companies should withdraw from Russia?

Participant A has answered ${e://Field/transferA2}

Participant B has answered ${e://Field/transferB2}

End of Block: Feedback screen attitude 1, 2, 3

Start of Block: Identity3

id3z I identify with the sample that my partner is representative of

- o Strongly disagree (1)
- o Disagree (2)
- o Somewhat disagree (3)
- o Neither agree nor disagree (4)

- Somewhat agree (5)
- Agree (6)
- Strongly agree (7)

id3b I feel solidarity with the sample

- Strongly disagree (1)
- Disagree (2)
- Somewhat disagree (3)
- Neither agree nor disagree (4)
- Somewhat agree (5)
- Agree (6)
- Strongly agree (7)

id3c I feel strong ties with the sample

- Strongly disagree (1)
- Disagree (2)
- Somewhat disagree (3)
- Neither agree nor disagree (4)
- Somewhat agree (5)
- Agree (6)
- Strongly agree (7)

End of Block: Identity3

Start of Block: Attitude 4

att5 Do you think that the majority of the news you consume about the war is unbiased, accurate and fact checked?

- o Yes (1)
- o No (2)

End of Block: Attitude 4

Start of Block: Send block 4

Q229

Page Break

Q230

End of Block: Send block 4

Start of Block: Get block 4

Q253

Waiting for ? participant(s).

Q254 Timing

First Click (1)

Last Click (2)

Page Submit (3)

Click Count (4)

Page Break

Q255

End of Block: Get block 4

Start of Block: Feedback screen 4

Q203
Do you think that the majority of the news you consume about the war is unbiased?

Participant A has answered ${e://Field/transferA5}

Participant B has answered ${e://Field/transferB5}

Previous attitudes:

1. 'Do you think your country of origin should extend unlimited welcome to as many refugees as need safe refuge?'

Participant A has answered ${e://Field/transferA1}

Participant B has answered ${e://Field/transferB1}

2. Do you think that companies should withdraw from Russia?

Participant A has answered ${e://Field/transferA2}

Participant B has answered ${e://Field/transferB2}

3. Do you think NATO’s expansion had a strong influence on Russia’s invasion of Ukraine?

Participant A has answered ${e://Field/transferA3}

Participant B has answered ${e://Field/transferB3}

End of Block: Feedback screen 4

Start of Block: Identity 4

id5a I identify with the sample that my partner is representative of

- o Strongly disagree (1)
- o Disagree (2)
- o Somewhat disagree (3)
- o Neither agree nor disagree (4)
- o Somewhat agree (5)
- o Agree (6)
- o Strongly agree (7)

id5b I feel solidarity with the sample

- o Strongly disagree (1)
- o Disagree (2)
- o Somewhat disagree (3)
- o Neither agree nor disagree (4)
- o Somewhat agree (5)
- o Agree (6)
- o Strongly agree (7)

id5c I feel strong ties with the sample

- o Strongly disagree (1)
- o Disagree (2)
- o Somewhat disagree (3)
- o Neither agree nor disagree (4)
- o Somewhat agree (5)
- o Agree (6)
- o Strongly agree (7)

End of Block: Identity 4

Start of Block: Attitude 5

att6 Do you think your country of residence should give money towards Ukrainian forces?

- o Yes (1)
- o No (2)

End of Block: Attitude 5

Start of Block: Send block 5

Q231

Page Break

Q232

End of Block: Send block 5

Start of Block: Get block 5

Q256

Waiting for ? participant(s).

Q257 Timing

First Click (1)

Last Click (2)

Page Submit (3)

Click Count (4)

Page Break

Q258

End of Block: Get block 5

Start of Block: Feedback screen 5

Q204
Do you think your country of origin should give money towards Ukrainian forces?

Participant A has answered ${e://Field/transferA6}

Participant B has answered ${e://Field/transferB6}

Previous attitudes:

1. 'Do you think your country of origin should extend unlimited welcome to as many refugees as

need safe refuge?'

Participant A has answered ${e://Field/transferA1}

Participant B has answered ${e://Field/transferB1}

2. Do you think that companies should withdraw from Russia?

Participant A has answered ${e://Field/transferA2}

Participant B has answered ${e://Field/transferB2}

3. Do you think NATO’s expansion had a strong influence on Russia’s invasion of Ukraine?

Participant A has answered ${e://Field/transferA3}

Participant B has answered ${e://Field/transferB3}

4. Do you think that the majority of the news you consume about the war is unbiased?

Participant A has answered ${e://Field/transferA5}

Participant B has answered ${e://Field/transferB5}

End of Block: Feedback screen 5

Start of Block: Identity 5

id6a I identify with the sample that my partner is representative of

- o Strongly disagree (1)
- o Disagree (2)
- o Somewhat disagree (3)
- o Neither agree nor disagree (4)
- o Somewhat agree (5)
- o Agree (6)
- o Strongly agree (7)

id6b I feel solidarity with the sample

- o Strongly disagree (1)
- o Disagree (2)
- o Somewhat disagree (3)
- o Neither agree nor disagree (4)
- o Somewhat agree (5)
- o Agree (6)
- o Strongly agree (7)

id6c I feel strong ties with the sample

- o Strongly disagree (1)
- o Disagree (2)
- o Somewhat disagree (3)
- o Neither agree nor disagree (4)
- o Somewhat agree (5)
- o Agree (6)
- o Strongly agree (7)

End of Block: Identity 5

Start of Block: Attitude 6

Att7 Do you think your country of residence should give arms/weapons to Ukrainian forces?

- o Yes (1)
- o No (2)

End of Block: Attitude 6

Start of Block: Send block 6

Q233

Page Break

Q234

End of Block: Send block 6

Start of Block: Get block 6

Q259

Waiting for ? participant(s).

Q260 Timing

First Click (1)

Last Click (2)

Page Submit (3)

Click Count (4)

Page Break

Q261

End of Block: Get block 6

Start of Block: Feedback screen 6

Q205
Do you think your country of residence should give arms/weapons to Ukrainian forces?

Participant A has answered ${e://Field/transferA7}

Participant B has answered ${e://Field/transferB7}

Previous attitudes:

1. 'Do you think your country of origin should extend unlimited welcome to as many refugees as need safe refuge?'

Participant A has answered ${e://Field/transferA1}

Participant B has answered ${e://Field/transferB1}

2. Do you think that companies should withdraw from Russia?

Participant A has answered ${e://Field/transferA2}

Participant B has answered ${e://Field/transferB2}

3. Do you think NATO’s expansion had a strong influence on Russia’s invasion of Ukraine?

Participant A has answered ${e://Field/transferA3}

Participant B has answered ${e://Field/transferB3}

4. Do you think that the majority of the news you consume about the war is unbiased?

Participant A has answered ${e://Field/transferA5}

Participant B has answered ${e://Field/transferB5}

5. Do you think your country of origin should give money towards Ukrainian forces?

Participant A has answered ${e://Field/transferA6}

Participant B has answered ${e://Field/transferB6}

End of Block: Feedback screen 6

Start of Block: Identity 6

id7a I identify with the sample that my partner is representative of

- Strongly disagree (1)
- Disagree (2)
- Somewhat disagree (3)
- Neither agree nor disagree (4)
- Somewhat agree (5)
- Agree (6)
- Strongly agree (7)

id7b I feel solidarity with the sample

- Strongly disagree (1)
- Disagree (2)
- Somewhat disagree (3)
- Neither agree nor disagree (4)
- Somewhat agree (5)
- Agree (6)
- Strongly agree (7)

id7c I feel strong ties with the sample

- Strongly disagree (1)
- Disagree (2)
- Somewhat disagree (3)
- Neither agree nor disagree (4)
- Somewhat agree (5)
- Agree (6)
- Strongly agree (7)

End of Block: Identity 6

Start of Block: Attitude 7

Att8 Do you think Ukraine should be offered entrance into NATO?

- o Yes (1)
- o No (2)

End of Block: Attitude 7

Start of Block: Send block 7

Q235

Page Break

Q236

End of Block: Send block 7

Start of Block: Get block 7

Q262

Waiting for ? participant(s).

Q263 Timing

First Click (1)

Last Click (2)

Page Submit (3)

Click Count (4)

Page Break

Q264

End of Block: Get block 7

Start of Block: Feedback screen 7

Q206
Do you think Ukraine should be offered entrance into NATO?

Participant A has answered ${e://Field/transferA8}

Participant B has answered ${e://Field/transferB8}

Previous attitudes:

1. 'Do you think your country of origin should extend unlimited welcome to as many refugees as need safe refuge?'

Participant A has answered ${e://Field/transferA1}

Participant B has answered ${e://Field/transferB1}

2. Do you think that companies should withdraw from Russia?

Participant A has answered ${e://Field/transferA2}

Participant B has answered ${e://Field/transferB2}

3. Do you think NATO's expansion had a strong influence on Russia's invasion of Ukraine?

Participant A has answered ${e://Field/transferA3}

Participant B has answered ${e://Field/transferB3}

4. Do you think that the majority of the news you consume about the war is unbiased?

Participant A has answered ${e://Field/transferA5}

Participant B has answered ${e://Field/transferB5}

5. Do you think your country of origin should give money towards Ukrainian forces?

Participant A has answered ${e://Field/transferA6}

Participant B has answered ${e://Field/transferB6}

6. Do you think your country of residence should give arms/weapons to Ukrainian forces?

Participant A has answered ${e://Field/transferA7}

Participant B has answered ${e://Field/transferB7}

End of Block: Feedback screen 7

Start of Block: Identity 7

id8a I identify with the sample that my partner is representative of

- o Strongly disagree (1)
- o Disagree (2)
- o Somewhat disagree (3)
- o Neither agree nor disagree (4)
- o Somewhat agree (5)
- o Agree (6)
- o Strongly agree (7)

id8b I feel solidarity with the sample

- o Strongly disagree (1)
- o Disagree (2)
- o Somewhat disagree (3)
- o Neither agree nor disagree (4)
- o Somewhat agree (5)
- o Agree (6)
- o Strongly agree (7)

id8c I feel strong ties with the sample

- o Strongly disagree (1)
- o Disagree (2)
- o Somewhat disagree (3)
- o Neither agree nor disagree (4)
- o Somewhat agree (5)
- o Agree (6)
- o Strongly agree (7)

End of Block: Identity 7

Start of Block: Attitude 8

Att9 Do you think your country of residence should send troops to fight alongside Ukrainian forces?

- o Yes (1)
- o No (2)

End of Block: Attitude 8

Start of Block: Send block 8

Q237

Page Break

Q238

End of Block: Send block 8

Start of Block: Get block 8

Q265

Waiting for ? participant(s).

Q266 Timing

First Click (1)

Last Click (2)

Page Submit (3)

Click Count (4)

Page Break

Q267

End of Block: Get block 8

Start of Block: Feedback screen 8

Q207
Do you think your country of residence should send troops to fight alongside Ukrainian forces?

Participant A has answered ${e://Field/transferA9}

Participant B has answered ${e://Field/transferB9}

Previous attitudes:

1. 'Do you think your country of origin should extend unlimited welcome to as many refugees as need safe refuge?'

Participant A has answered ${e://Field/transferA1}

Participant B has answered ${e://Field/transferB1}

2. ' Do you think that companies should withdraw from Russia?

Participant A has answered ${e://Field/transferA2}

Participant B has answered ${e://Field/transferB2}

3. Do you think NATO's expansion had a strong influence on Russia's invasion of Ukraine?

Participant A has answered ${e://Field/transferA3}

Participant B has answered ${e://Field/transferB3}

4. Do you think that the majority of the news you consume about the war is unbiased?

Participant A has answered ${e://Field/transferA5}

Participant B has answered ${e://Field/transferB5}

5. Do you think your country of origin should give money towards Ukrainian forces?

Participant A has answered ${e://Field/transferA6}

Participant B has answered ${e://Field/transferB6}

6. Do you think your country of residence should give arms/weapons to Ukrainian forces?

Participant A has answered ${e://Field/transferA7}

Participant B has answered ${e://Field/transferB7}

7. Do you think Ukraine should be offered entrance into NATO?

Participant A has answered ${e://Field/transferA8}

Participant B has answered ${e://Field/transferB8}

End of Block: Feedback screen 8

Start of Block: Identity 8

id9a I identify with the sample that my partner is representative of

- o Strongly disagree (1)
- o Disagree (2)
- o Somewhat disagree (3)
- o Neither agree nor disagree (4)
- o Somewhat agree (5)
- o Agree (6)

- Strongly agree (7)

id9b I feel solidarity with the sample

- Strongly disagree (1)
- Disagree (2)
- Somewhat disagree (3)
- Neither agree nor disagree (4)
- Somewhat agree (5)
- Agree (6)
- Strongly agree (7)

id9c I feel strong ties with the sample

- Strongly disagree (1)
- Disagree (2)
- Somewhat disagree (3)
- Neither agree nor disagree (4)
- Somewhat agree (5)
- Agree (6)
- Strongly agree (7)

End of Block: Identity 8

Start of Block: Attitude 9

Att10 Do you think your country of residence should refuse to engage with Russia for as long as Putin remains president?

- Yes (1)

o No (2)

End of Block: Attitude 9

Start of Block: Send block 9

Q239

Page Break

Q240

End of Block: Send block 9

Start of Block: Get block 9

Q268

Waiting for ? participant(s).

Q269 Timing

First Click (1)

Last Click (2)

Page Submit (3)

Click Count (4)

Page Break

Q270

End of Block: Get block 9

Start of Block: Feedback screen 9

Q208
Do you think your country should refuse to engage with Russia for as long as Putin remains president?

Participant A has answered ${e://Field/transferA10}

Participant B has answered ${e://Field/transferB10}

Previous attitudes:

1. 'Do you think your country of origin should extend unlimited welcome to as many refugees as need safe refuge?'

Participant A has answered ${e://Field/transferA1}

Participant B has answered ${e://Field/transferB1}

2. Do you think that companies should withdraw from Russia?

Participant A has answered ${e://Field/transferA2}

Participant B has answered ${e://Field/transferB2}

3. Do you think NATO's expansion had a strong influence on Russia's invasion of Ukraine?

Participant A has answered ${e://Field/transferA3}

Participant B has answered ${e://Field/transferB3}

4. Do you think that the majority of the news you consume about the war is unbiased?

Participant A has answered ${e://Field/transferA5}

Participant B has answered ${e://Field/transferB5}

5. Do you think your country of origin should give money towards Ukrainian forces?

Participant A has answered ${e://Field/transferA6}

Participant B has answered ${e://Field/transferB6}

6. Do you think your country of residence should give arms/weapons to Ukrainian forces?

Participant A has answered ${e://Field/transferA7}

Participant B has answered ${e://Field/transferB7}

7. Do you think Ukraine should be offered entrance into NATO?

Participant A has answered ${e://Field/transferA8}

Participant B has answered ${e://Field/transferB8}

8. Do you think your country of residence should send troops to fight alongside Ukrainian forces?

Participant A has answered ${e://Field/transferA9}

Participant B has answered ${e://Field/transferB9}

End of Block: Feedback screen 9

Start of Block: Identity 9

id10a I identify with the sample that my partner is representative of

- o Strongly disagree (1)
- o Disagree (2)
- o Somewhat disagree (3)
- o Neither agree nor disagree (4)
- o Somewhat agree (5)
- o Agree (6)
- o Strongly agree (7)

id10b I feel solidarity with the sample

- o Strongly disagree (1)
- o Disagree (2)
- o Somewhat disagree (3)
- o Neither agree nor disagree (4)

- Somewhat agree (5)
- Agree (6)
- Strongly agree (7)

id10c I feel strong ties with the sample

- Strongly disagree (1)
- Disagree (2)
- Somewhat disagree (3)
- Neither agree nor disagree (4)
- Somewhat agree (5)
- Agree (6)
- Strongly agree (7)

End of Block: Identity 9

Start of Block: Attitude 10

att11
Social media sites such as instagram and facebook made exceptions to their policy against inciting violence, so long as the posts represented political expression against Russian forces invading Ukraine.

Social media sites made the right decision to allow these posts inciting violence:

- Agree (1)
- Disagree (2)

End of Block: Attitude 10

Start of Block: Send block 10

Q241

Page Break

Q242

End of Block: Send block 10

Start of Block: Get block 10

Q271

Waiting for ? participant(s).

Q272 Timing

First Click (1)

Last Click (2)

Page Submit (3)

Click Count (4)

Page Break

Q273

End of Block: Get block 10

Start of Block: Feedback screen 10

Q209 Social media sites made the right decision to allow these posts inciting violence:

Participant A has answered ${e://Field/transferA11}

Participant B has answered ${e://Field/transferB11}

Previous attitudes:

1. 'Do you think your country of origin should extend unlimited welcome to as many refugees as need safe refuge?'

Participant A has answered ${e://Field/transferA1}

Participant B has answered ${e://Field/transferB1}

2. Do you think that companies should withdraw from Russia?

Participant A has answered ${e://Field/transferA2}

Participant B has answered ${e://Field/transferB2}

3. Do you think NATO's expansion had a strong influence on Russia's invasion of Ukraine?

Participant A has answered ${e://Field/transferA3}

Participant B has answered ${e://Field/transferB3}

4. Do you think that the majority of the news you consume about the war is unbiased?

Participant A has answered ${e://Field/transferA5}

Participant B has answered ${e://Field/transferB5}

5. Do you think your country of origin should give money towards Ukrainian forces?

Participant A has answered ${e://Field/transferA6}

Participant B has answered ${e://Field/transferB6}

6. Do you think your country of residence should give arms/weapons to Ukrainian forces?

Participant A has answered ${e://Field/transferA7}

Participant B has answered ${e://Field/transferB7}

7. Do you think Ukraine should be offered entrance into NATO?

Participant A has answered ${e://Field/transferA8}

Participant B has answered ${e://Field/transferB8}

8. Do you think your country of residence should send troops to fight alongside Ukrainian

forces?

Participant A has answered ${e://Field/transferA9}

Participant B has answered ${e://Field/transferB9}

9. Do you think your country should refuse to engage with Russia for as long as Putin remains president?

Participant A has answered ${e://Field/transferA10}

Participant B has answered ${e://Field/transferB10}

End of Block: Feedback screen 10

Start of Block: Identity 10

id11a I identify with the sample that my partner is representative of

- o Strongly disagree (1)
- o Disagree (2)
- o Somewhat disagree (3)
- o Neither agree nor disagree (4)
- o Somewhat agree (5)
- o Agree (6)
- o Strongly agree (7)

id11b I feel solidarity with the sample

- o Strongly disagree (1)
- o Disagree (2)
- o Somewhat disagree (3)

- Neither agree nor disagree (4)
- Somewhat agree (5)
- Agree (6)
- Strongly agree (7)

id11c I feel strong ties with the sample

- Strongly disagree (1)
- Disagree (2)
- Somewhat disagree (3)
- Neither agree nor disagree (4)
- Somewhat agree (5)
- Agree (6)
- Strongly agree (7)

End of Block: Identity 10

Start of Block: Attitude 11

Att12
The current situation is that western Europe needs to import 75% of its oil and 50% of its gas, and Russia fulfils most of this requirement.

Do you think that your countries should refuse to buy oil/gas from Russia, knowing that this may lead to inflation in fuel prices or oil/gas shortages?

- Yes (1)
- No (2)

End of Block: Attitude 11

Start of Block: Send block 11

Q243

Page Break

Q244

End of Block: Send block 11

Start of Block: Get block 11

Q274

Waiting for ? participant(s).

Q275 Timing

First Click (1)

Last Click (2)

Page Submit (3)

Click Count (4)

Page Break

Q276

End of Block: Get block 11

Start of Block: Feedback screen 11

Q210
Do you think that your country of residence should refuse to buy oil/gas from Russia, knowing that this may lead to inflation in fuel prices or oil/gas shortages in your country?

Participant A has answered ${e://Field/transferA12}

Participant B has answered ${e://Field/transferB12}

Previous attitudes:

1. 'Do you think your country of origin should extend unlimited welcome to as many refugees as need safe refuge?'

Participant A has answered ${e://Field/transferA1}

Participant B has answered ${e://Field/transferB1}

2. Do you think that companies should withdraw from Russia?

Participant A has answered ${e://Field/transferA2}

Participant B has answered ${e://Field/transferB2}

3. Do you think NATO's expansion had a strong influence on Russia's invasion of Ukraine?

Participant A has answered ${e://Field/transferA3}

Participant B has answered ${e://Field/transferB3}

4. Do you think that the majority of the news you consume about the war is unbiased?

Participant A has answered ${e://Field/transferA5}

Participant B has answered ${e://Field/transferB5}

5. Do you think your country of origin should give money towards Ukrainian forces?

Participant A has answered ${e://Field/transferA6}

Participant B has answered ${e://Field/transferB6}

6. Do you think your country of residence should give arms/weapons to Ukrainian forces?
Participant A has answered ${e://Field/transferA7}

Participant B has answered ${e://Field/transferB7}

7. Do you think Ukraine should be offered entrance into NATO?

Participant A has answered ${e://Field/transferA8}

Participant B has answered ${e://Field/transferB8}

8. Do you think your country of residence should send troops to fight alongside Ukrainian

forces?

Participant A has answered ${e://Field/transferA9}

Participant B has answered ${e://Field/transferB9}

9. Do you think your country should refuse to engage with Russia for as long as Putin remains president?

Participant A has answered ${e://Field/transferA10}

Participant B has answered ${e://Field/transferB10}

10. Social media sites such as instagram and facebook made exceptions to their policy against inciting violence, so long as the posts represented political expression against Russian forces invading Ukraine.
Social media sites made the right decision to allow these posts inciting violence:

Participant A has answered ${e://Field/transferA11}

Participant B has answered ${e://Field/transferB11}

End of Block: Feedback screen 11

Start of Block: Identity 11

id12a I identify with the sample that my partner is representative of

- o Strongly disagree (1)
- o Disagree (2)
- o Somewhat disagree (3)
- o Neither agree nor disagree (4)
- o Somewhat agree (5)
- o Agree (6)
- o Strongly agree (7)

id12b I feel solidarity with the sample

- o Strongly disagree (1)
- o Disagree (2)
- o Somewhat disagree (3)
- o Neither agree nor disagree (4)
- o Somewhat agree (5)
- o Agree (6)
- o Strongly agree (7)

id12c I feel strong ties with the sample

- o Strongly disagree (1)
- o Disagree (2)
- o Somewhat disagree (3)
- o Neither agree nor disagree (4)
- o Somewhat agree (5)
- o Agree (6)
- o Strongly agree (7)

End of Block: Identity 11

Start of Block: Superordinate attitude identification

Q242 Please think about other people in society who support Ukraine.

Superordinateidentif I identify **with other people in society** who support Ukraine

- o Strongly disagree (1)
- o Disagree (2)

- Somewhat disagree (3)
- Neither agree nor disagree (4)
- Somewhat agree (5)
- Agree (6)
- Strongly agree (7)

SuperordinateStrong I feel strong ties **with other people in society** who support Ukraine

- Strongly disagree (1)
- Disagree (2)
- Somewhat disagree (3)
- Neither agree nor disagree (4)
- Somewhat agree (5)
- Agree (6)
- Strongly agree (7)

SuperordinateSolidar I feel solidarity **with other people in society** who support Ukraine

- Strongly disagree (1)
- Disagree (2)
- Somewhat disagree (3)
- Neither agree nor disagree (4)
- Somewhat agree (5)
- Agree (6)
- Strongly agree (7)

End of Block: Superordinate attitude identification

Start of Block: Activism orientation scale

Q153
Think about the current crisis in Ukraine. How willing are you to

SignPetitions
Sign petitions in solidarity with Ukraine?

- o Extremely unlikely (1)
- o Unlikely (2)
- o Likely (3)
- o Extremely likely (4)

Share
Share promotion material on your social media accounts?

- o Extremely unlikely (1)
- o Unlikely (2)
- o Likely (3)
- o Extremely likely (4)

Q117 Display a poster or bumper sticker?

- o Extremely unlikely (1)
- o Unlikely (2)
- o Likely (3)
- o Extremely likely (4)

Q118 Invite a friend to attend a meeting or event in relation to this issue?

- o Extremely unlikely (1)

- o Unlikely (2)
- o Likely (3)
- o Extremely likely (4)

Q119 Purchase a poster, t-shirt, etc. that endorses this point of view?

- o Extremely unlikely (1)
- o Unlikely (2)
- o Likely (3)
- o Extremely likely (4)

Q122 Attend an informational meeting in relation to this issue?

- o Extremely unlikely (1)
- o Unlikely (2)
- o Likely (3)
- o Extremely likely (4)

Q123 Organize an event (e.g. talk, support group, march)?

- o Extremely unlikely (1)
- o Unlikely (2)
- o Likely (3)
- o Extremely likely (4)

Q124 Give a talk or lecture about this issue?

- o Extremely unlikely (1)
- o Unlikely (2)

- Likely (3)
- Extremely likely (4)

Q125 Go out of your way to collect information about this issue?

- Extremely unlikely (1)
- Unlikely (2)
- Likely (3)
- Extremely likely (4)

Q126 Campaign door-to-door about this issue?

- Extremely unlikely (1)
- Unlikely (2)
- Likely (3)
- Extremely likely (4)

Q127 Present facts to contest another person's views on this issue?

- Extremely unlikely (1)
- Unlikely (2)
- Likely (3)
- Extremely likely (4)

Q128 Donate money in relation to this issue?

- Extremely unlikely (1)
- Unlikely (2)
- Likely (3)

- Extremely likely (4)

Q131 Send a letter or e-mail expressing this opinion to the editor of a periodical or television show?

- Extremely unlikely (1)
- Unlikely (2)
- Likely (3)
- Extremely likely (4)

Q134 Confront jokes, statements, or innuendoes that oppose your opinion?

- Extremely unlikely (1)
- Unlikely (2)
- Likely (3)
- Extremely likely (4)

Q135 Boycott a product that opposes this view?

- Extremely unlikely (1)
- Unlikely (2)
- Likely (3)
- Extremely likely (4)

Q136 Distribute information representing this cause?

- Extremely unlikely (1)
- Unlikely (2)
- Likely (3)

o Extremely likely (4)

Q138 Send a letter or email about this issue to a public official?

o Extremely unlikely (1)
o Unlikely (2)
o Likely (3)
o Extremely likely (4)

Q143 Try to change a friend's or acquaintance's mind about this issue?

o Extremely unlikely (1)
o Unlikely (2)
o Likely (3)
o Extremely likely (4)

Q146 Try to change a relative's mind about this issue?

o Extremely unlikely (1)
o Unlikely (2)
o Likely (3)
o Extremely likely (4)

Q147 Wear a t-shirt or button displaying this group's message?

o Extremely unlikely (1)
o Unlikely (2)
o Likely (3)
o Extremely likely (4)

Q148 Keep track of the views of members of Congress regarding this issue?

- o Extremely unlikely (1)
- o Unlikely (2)
- o Likely (3)
- o Extremely likely (4)

Q149 Participate in discussion groups designed to discuss this issue and solutions?

- o Extremely unlikely (1)
- o Unlikely (2)
- o Likely (3)
- o Extremely likely (4)

Q150 Campaign by phone regarding this issue?

- o Extremely unlikely (1)
- o Unlikely (2)
- o Likely (3)
- o Extremely likely (4)

End of Block: Activism orientation scale

Start of Block: Country

Q210 Please enter your current country of residence below:

____

Q211 If different from the previous entry, please enter your country of birth below:

____

End of Block: Country

Start of Block: COMPLETE BLOCK NR

complete
Thank you for your time. This survey is complete.

Q431 End of Study Teamwork & Memory Recall (Ethics Code: 2019_06_19_EHS)
Thank you for your participation.
Your participation is greatly appreciated and helps us in our research.
Study Explanation
In this study, we investigated group interaction and attitudes. Participants in this study answered a questions regarding their feelings towards attitude statements and arbitrary information questions. Participants chatted with other participants and shared answers to the arbitrary information questions. Following this every participant answered questions relating to group identity and attitude strength. No aspect of the study tested memory recall. That was a cover story given to all participants at the beginning so that their behaviour would not be influenced by the expectations of the experimenter. By participating in this study, you have experienced how social psychological research is done. Procedures such as those used in the current study are of great importance for this research. Also, if you think of conducting research yourself in your career or as part of your study, this experiment may give you insights in how you can put this into practice. If you have further questions or wish to get more in-depth information on a specific topic, you can contact the researcher at X.

End of Block: COMPLETE BLOCK NR
