# Supplementary material for: Strategic attitude expressions as identity performance and identity creation in interaction
Source: Commun Psychol. 2024 Apr 8;2:27. doi: 10.1038/s44271-024-00076-7 (PMC11331991; doi:10.1038/s44271-024-00076-7)
Supplement: Supplementary file 3 — Reporting Summary [file 44271_2024_76_MOESM3_ESM.pdf]

nature portfolio

Corresponding author(s): DBPR COMMSPSYCHOL-23-0078B

Last updated by author(s): 22/01/24

Reporting Summary

Nature Portfolio wishes to improve the reproducibility of the work that we publish. This form provides structure for consistency and transparency in reporting. For further information on Nature Portfolio policies, see our Editorial Policies and the Editorial Policy Checklist.

Statistics

For all statistical analyses, confirm that the following items are present in the figure legend, table legend, main text, or Methods section.

n/a	Confirmed	
☐	☒	The exact sample size (*n*) for each experimental group/condition, given as a discrete number and unit of measurement
☐	☒	A statement on whether measurements were taken from distinct samples or whether the same sample was measured repeatedly
☐	☒	The statistical test(s) used AND whether they are one- or two-sided<br>*Only common tests should be described solely by name; describe more complex techniques in the Methods section.*
☐	☒	A description of all covariates tested
☐	☒	A description of any assumptions or corrections, such as tests of normality and adjustment for multiple comparisons
☐	☒	A full description of the statistical parameters including central tendency (e.g. means) or other basic estimates (e.g. regression coefficient) AND variation (e.g. standard deviation) or associated estimates of uncertainty (e.g. confidence intervals)
☐	☒	For null hypothesis testing, the test statistic (e.g. *F*, *t*, *r*) with confidence intervals, effect sizes, degrees of freedom and *P* value noted<br>*Give P values as exact values whenever suitable.*
☒	☐	For Bayesian analysis, information on the choice of priors and Markov chain Monte Carlo settings
☐	☒	For hierarchical and complex designs, identification of the appropriate level for tests and full reporting of outcomes
☐	☒	Estimates of effect sizes (e.g. Cohen's *d*, Pearson's *r*), indicating how they were calculated

Our web collection on statistics for biologists contains articles on many of the points above.

Software and code

Policy information about availability of computer code

Data collection	On September 19th, 2022, data were collected via Prolific (version 2022) using Qualtrics (Version 2022).<br><br>References<br>Prolific (2014 – 2023). Prolific (Version 2022). London, UK. https://www.prolific.co<br>Qualtrics (2005 - 2023). Qualtrics (Version 2022). Provo, Utah, USA. https://www.qualtrics.com
Data analysis	All data and analytic code are available (https://osf.io/w8shc/?view_only=34d38b51dc8a4691bf4fec2dbe3cde66)). Data was analysed using SPSS version 26, R, and Python.

For manuscripts utilizing custom algorithms or software that are central to the research but not yet described in published literature, software must be made available to editors and reviewers. We strongly encourage code deposition in a community repository (e.g. GitHub). See the Nature Portfolio guidelines for submitting code & software for further information.

Data

Policy information about availability of data

All manuscripts must include a data availability statement. This statement should provide the following information, where applicable:

- Accession codes, unique identifiers, or web links for publicly available datasets
- A description of any restrictions on data availability
- For clinical datasets or third party data, please ensure that the statement adheres to our policy

· All data and analytic code are available (https://osf.io/w8shc/?view_only=34d38b51dc8a4691bf4fec2dbe3cde66).

Human research participants

Policy information about studies involving human research participants and Sex and Gender in Research.

Reporting on sex and gender	There were 1368 participants ... Of these, 722 self-identified as female, 639 as male and 7 as non-binary.<br><br>Gender based analysis was not reported as we did not have gender-based research questions.
Population characteristics	After preregistered exclusions , there were 1368 participants ranging in age from 18 to 75 (M = 36.07, SD = 12.27).
Recruitment	Prior to data collection, individuals who had participated in our previous studies were precluded (to avoid practice effects). The survey was made available to individuals whose first language was English, who had a prolific approval rate greater than 94, who had a minimum of 50 prior survey submissions, and who were currently located in Europe . Prolific samples are susceptible to several biases including first come first serve response bias, WEIRD bias, selection bias, and reward per hour bias (see Prolific Team, 2023 for more detail). Nonetheless, crowdsourced data has been found to be of high quality (Buhrmester et al., 2011) and Prolific data has been found to have higher quality of data than other crowd sourcing platforms (Peer et al., 2021), it allows us to gather data from wide geographical areas, and data seems to function similarly well to laboratory studies (Peer et al., 2017).
Ethics oversight	This research study adheres to the ethical guidelines specified in the APA code of conduct as well as authors' national ethics guidelines. The study has received ethical approval from the University of Limerick, Education and Health Sciences Research Ethics Committee (19_06_2019). All research is conducted ethically, results are reported honestly, and the submitted work is original and not plagiarised (Wiley Author Guidelines, 2022).

Note that full information on the approval of the study protocol must also be provided in the manuscript.

Field-specific reporting

Please select the one below that is the best fit for your research. If you are not sure, read the appropriate sections before making your selection.

☐ Life sciences ☒ Behavioural & social sciences ☐ Ecological, evolutionary & environmental sciences

For a reference copy of the document with all sections, see nature.com/documents/nr-reporting-summary-flat.pdf

Behavioural & social sciences study design

All studies must disclose on these points even when the disclosure is negative.

Study description	In our first study we use real-world data to assess whether clusters of followers online tend to share similar attitudes to one another. Specifically, we gathered tweets from Twitter based on shared hashtags related to the Ukraine Russia crisis which escalated in 2022 with the Russian invasion of Ukraine. We captured a followership network, ran community detection algorithms to identify groups, and assessed the most popular hashtags shared by each follower community to visualise how attitude alignment occurs in the wild.<br><br>In study two we use controlled, interactive, online experimental methods to quantitatively explore motivated attitude alignment.
Research sample	We searched twitter to identify popular hashtags related to the ongoing invasion of the Ukraine, as well as hashtags that were 'trending' at the time (see supplementary materials for full list of hashtags used), attempting to identify as many dimensions of the online discourse as possible. We then identified all users who had tweeted or retweeted these hashtags between February and June 2022 (from Russia's invasion of Ukraine and for five months after this) and built a followership network of these users, where users are linked to every user they are followed by, if that user also has tweeted or retweeted one of the hashtags. The followership network contained 8149 users.<br><br>Study two<br><br>On September 19th, 2022, data were collected via Prolific (version 2022) using Qualtrics (Version 2022). Prior to data collection,

individuals who had participated in our previous studies were precluded (to avoid practice effects). The survey was made available to individuals whose first language was English, who had a prolific approval rate greater than 94, who had a minimum of 50 prior survey submissions, and who were currently located in Europe..... Prolific data has been found to have higher quality of data than other crowd sourcing platforms (Peer et al., 2021), it allows us to gather data from wide geographical areas, and data seems to function similarly well to laboratory studies (Peer et al., 2017).

Sampling strategy

Study 2
Data was collected on Prolific using convenience sampling. We conducted a sensitivity power analysis (Faul et al., 2017) with Anova (fixed effects one way) as the statistical test. Based on N = 1368, with alpha significance criterion .05 two-tailed and a standard power criterion of 80%, the analysis had 80% power to detect an effect size of f = .08 (equivalent to d = .16).

Data collection

Study one
We searched twitter to identify popular hashtags related to the ongoing invasion of the Ukraine, as well as hashtags that were 'trending' at the time (see supplementary materials for full list of hashtags used), attempting to identify as many dimensions of the online discourse as possible. We then identified all users who had tweeted or retweeted these hashtags between February and June 2022 (from Russia's invasion of Ukraine and for five months after this) and built a followership network of these users, where users are linked to every user they are followed by, if that user also has tweeted or retweeted one of the hashtags. The followership network contained 8149 users.

Study 2
On September 19th, 2022, data were collected via Prolific (version 2022) using Qualtrics (Version 2022). The exact survey can be found in the supplementary materials.

Timing

Study one
We scraped Twitter for tweets in English containing hashtags relating to the Russia/Ukraine war, starting from the beginning of the invasion (24/02/22) and ending the day we commenced gathering data (28/06/22).

Study two
On September 19th, 2022, data were collected via Prolific (version 2022) using Qualtrics (Version 2022).

Data exclusions

Study one
We then examined the frequencies of hashtag expressions for each tweet and chose six hashtags that had high tweet or retweet frequencies. Our final sample consisted of a subset of users who had tweeted one of these hashtags (Supplementary Note 4). We then created a dataset of all tweets in the original dataset from users who were in our final sample. Next, we gathered the follower list for each of these users, discarding the data for any user whose account information was unavailable through the Twitter API (e.g., restricted, banned, or deleted accounts). We also discarded any user in the follower lists for whom we had no tweets. This left us with 8,149 users.

Study 2
After preregistered exclusions there were 1368 participants.
Those who did not give consent (N = 1), who progressed less than 95% in the study (N = 0), who had less than two people in their dyads (N = 27), and who give the incorrect response to
"What is your letter?" (N = 9) were removed from analyses.

Non-participation

Study 2
289 participants returned their submissions without completing them. It is unclear why these participants began the study but did not finish it.

Randomization

Study 2
The current study has both an experimental and quasi experimental design (see table 3). The experimental manipulation separated participants into the experimental condition who observed each other's attitude answers, and the control condition who observed each other's answers to the arbitrary non-attitude information questions. The quasi-experimental element involved further subdivision of the control and experimental groups based on their attitude congruence on the first attitude, into; experimental ingroup (those who are aware they have attitude congruence on the first attitude); experimental outgroup (those who are aware they have attitude incongruence on the first attitude); control ingroup (those who had attitude congruence on the first attitude but were unaware of this congruence); and control outgroup (those who had attitude incongruence on the first attitude but were unaware of this incongruence) . Participants were not explicitly told they had been categorised into a group.

Reporting for specific materials, systems and methods

We require information from authors about some types of materials, experimental systems and methods used in many studies. Here, indicate whether each material, system or method listed is relevant to your study. If you are not sure if a list item applies to your research, read the appropriate section before selecting a response.

Materials & experimental systems

n/a	Involved in the study
☒	☐ Antibodies
☒	☐ Eukaryotic cell lines
☒	☐ Palaeontology and archaeology
☒	☐ Animals and other organisms
☒	☐ Clinical data
☒	☐ Dual use research of concern

Methods

n/a	Involved in the study
☒	☐ ChIP-seq
☒	☐ Flow cytometry
☒	☐ MRI-based neuroimaging
